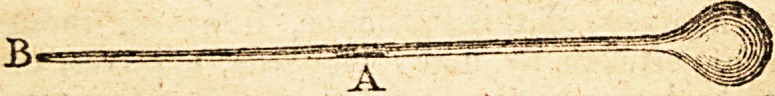# Mr. Giraud's Mode of Preserving Vaccine Matter

**Published:** 1803-05-01

**Authors:** John Thomas Giraud

**Affiliations:** Feversham


### *To the Editors of the Medical and Physical Journal.*

GENTLEMEN,

AMONG your numerous Correspondents on the subject of the Vaccine Inoculation, I do not find that any of them have hit upon a method which I have for some time made use of, for preserving the matter in an active state, and conveying it to any distance. I think my method claims a superiority over any other that I have heard of, or seen described, as by it the matter is preserved in a fluid state, and absolutely excluded from contact with the air; by which means it is less likely to undergo a decomposition, and its activity not so liable to be destroyed as when it is suffered to dry, and to be exposed to the access of air.

My method is as follows. I make use of a small glass bulb, with a shank or tube to it, of the size and form of the under impression,

Viz. I select a subject with a full pustule, in which I make a small puncture; in a few seconds a small drop of matter is to be seen on this puncture; I then dip my glass bulb into a cup of boiling water, holding it therein long enough for the expanded air to escape; I then take it out and instantly apply the orifice of the tube to the drop of matter, which will be drawn into the tube as the bulb cools; I then immediately seal the tube hermetically by holding the end of it in the flame of a candle.

To

To make use of the matter thus preserved, the extreme end of the tube must be broken, and the point of a lancet applied to it; at the same time, by gently approaching the bulb to the flame of a candle, the matter will by the expansion of the air be driven out and received on the point of the lancet. Or a puncture may be made on the arm, into which the end of the tube may be inserted, and then the candle gently applied to the bulb.

Vaccine matter preserved by this method has been inserted, at various periods, from one week to upwards of two months, with complete success.

*Feversham,* I am, &c.

*April* 10, 1803. JOHN THOMAS GIRAUD.

A. A small portion of matter within the tube.
B. The end of the tube hermetically sealed.

P. S. Since writing the above I have examined the arm of a child inoculated on Thursday, the 7th instant, from one of my tubes armed on November 12, 1802, and have no doubt of its having taken.

## Figures and Tables

**Figure f1:**